# Prevalence and treatment of perinatal anxiety: diagnostic interview study

**DOI:** 10.1192/bjo.2024.823

**Published:** 2024-12-13

**Authors:** Susan Ayers, Andrea Sinesi, Rose Meade, Helen Cheyne, Margaret Maxwell, Catherine Best, Stacey McNicol, Louise R. Williams, Una Hutton, Grace Howard, Judy Shakespeare, Fiona Alderdice, Julie Jomeen, the MAP Study Team

**Affiliations:** Centre for Maternal and Child Health Research, School of Health and Psychological Sciences, City, University of London, UK; Nursing, Midwifery and Allied Health Professions Research Unit, University of Stirling, UK; Midwifery Department, King's College London, UK; Retired General Practitioner, UK; National Perinatal Epidemiology Unit, Nuffield Department of Population Health, University of Oxford, UK; Faculty of Health, Southern Cross University, Australia

**Keywords:** Anxiety or fear-related disorders, out-patient treatment, perinatal psychiatry, psychological treatments, observational study

## Abstract

**Background:**

Anxiety affects around one in five women during pregnancy and after birth. However, there is no systematic information on the proportion of women with perinatal anxiety disorders who want or receive treatment.

**Aims:**

To examine (a) the prevalence of anxiety disorders during pregnancy and after birth in a population-based sample, and (b) the proportion of women with anxiety disorders who want treatment and receive treatment.

**Method:**

This study conducted 403 diagnostic interviews in early pregnancy (*n* = 102), mid-pregnancy (*n* = 99), late pregnancy (*n* = 102) or postpartum (*n* = 100). Participants also completed self-report measures of previous/current mental health problems and desire for treatment at every time point.

**Results:**

The prevalence of anxiety disorders over all time points combined was 19.9% (95% CI 16.1–24.1), with greatest prevalence in early pregnancy (25.5%, 95% CI 17.4–35.1). The most prevalent disorders were obsessive–compulsive disorder (8.2%, 95% CI 5.7–11.3) and generalised anxiety disorder (5.7%, 95% CI 3.7–8.4). The majority of women with anxiety disorders did not want professional help or treatment (79.8%). Most women with anxiety disorders who did want treatment (20.2%) were receiving treatment. The majority of participants with anxiety disorders had a history of mental health problems (64.6%).

**Conclusions:**

Prevalence rates overall are consistent with previous research, lending validity to the findings. However, findings challenge the assumption that everyone with a psychological disorder wants treatment. These findings highlight the importance of relationship-based care, where individual needs and contextual barriers to treatment can be explored.

Perinatal anxiety is thought to affect around one in five women, but prevalence estimates vary.^1,2^ Meta-analyses suggest moderate to severe anxiety symptoms affect around 23% of women in pregnancy and 15% after birth.^3^ Similarly, meta-analytic estimates of the prevalence of anxiety disorders vary from 21% overall^4^ to 15% in pregnancy and 10% after birth.^3^ There are also indications that perinatal mental health problems might be increasing, with indirect evidence from epidemiological studies that rates of maternal mental illness increased in recent years in the UK.^5,6^

Identifying and treating perinatal anxiety (symptoms and disorders) is important because anxiety may have an adverse impact on mothers and infants. Studies of the developmental origins of health and disease suggest intergenerational transmission of psychopathology from mothers to infants may occur through epigenetic modification of physiological stress response mechanisms, parenting and wider social and contextual factors.^7,8^ Anxiety in pregnancy is also associated with increased risk of preterm birth, low birth weight and poor infant emotional development,^9,10^ although it is unclear how much of these are attributable to anxiety or other confounding factors, such as pharmacotherapy or parenting styles.^1,8^

## Treatment of perinatal anxiety

Although there is evidence for potential benefits of treatments such as cognitive–behavioural therapy in reducing symptoms of anxiety,^11^ it is not clear that screening and treatment necessarily reduce long-term adverse outcomes for women and their infants.^12^ At present, it is not known how many women with perinatal anxiety disorders in the general population seek or receive treatment. Estimates from the literature on perinatal depression are that 30–50% of women with perinatal mental health problems are identified and <10% are referred to specialist care.^13^ The current research therefore aimed to determine (a) the prevalence of anxiety disorders during pregnancy and after birth in a UK population-based sample and (b) the proportion of women with anxiety disorders who wanted and received treatment. Anxiety disorders are often comorbid with depression, so major depressive disorder was also examined. We use terms such as ‘women’, ‘maternity’ etc. throughout this paper to refer to those who are pregnant and give birth. We acknowledge that not all people who are pregnant and give birth identify as women.

## Method

### Study design

We conducted a diagnostic interview study of 403 participants drawn from the Methods of Assessing Perinatal Anxiety (MAP) cohort of women who completed self-assessment measures in early pregnancy (mean 11.4 weeks, s.d. 2.0), mid-pregnancy (mean 23.0 weeks, s.d. 1.3), late pregnancy (mean 31.9 weeks, s.d. 1.2) and postpartum (mean 7.9 weeks, s.d. 2.4). The MAP study was pre-registered (reference: researchregistry5980)^14^ and the protocol is available online.^15^

### Ethical approvals

The authors assert that all procedures contributing to this work comply with the ethical standards of the relevant national and institutional committees on human experimentation and with the Helsinki Declaration of 1975, as revised in 2013. All procedures involving human patients were approved by the National Health Service West of Scotland Research Ethics Committee (approval number 20/WS/0065), Health Research Authority (approval number IRAS 274901) and City, University of London (approval number ETH1920-0572).

### Sample

Participants were drawn from the MAP cohort of 2243 pregnant women recruited through 12 National Health Service (NHS) Trusts in England and five NHS Health Boards in Scotland. Women were eligible for the MAP cohort if they were aged 16 years or over, <15 weeks pregnant at the time of recruitment, able to provide written informed consent and had sufficient English to complete the questionnaires. Participants for the diagnostic interview sample were drawn consecutively from the MAP cohort at each time point. Consecutive sampling was used to minimise bias,^16^ and a 10:1 ratio of participants from England and Scotland was achieved, which reflects relative annual births for the two nations. Participants were sampled at each time point (early pregnancy *n* = 102, mid-pregnancy *n* = 99, late pregnancy *n* = 102, postpartum *n* = 100), with each participant interviewed at one time point only. Sample size calculations were based on an estimated prevalence of 15% of women experiencing clinically significant anxiety in the perinatal period.^3^ Participants were recruited and interviewed from January 2021 to April 2022.

### Measures

Diagnostic interviews were conducted using a gold standard interview for psychiatric disorders: the Mini-International Neuropsychiatric Interview version 7.0.2 (MINI),^17^ which assesses anxiety disorders according to the DSM-5.^18^ Modules administered were panic disorder, agoraphobia, social anxiety disorder, obsessive–compulsive disorder (OCD), post-traumatic stress disorder, generalised anxiety disorder (GAD), specific phobia and major depressive episode. Disorders were recorded as present if participants currently met diagnostic criteria.

Treatment was measured by self-report questionnaire at each time point. Participants were asked whether they were currently experiencing psychological or mental health problems, and this was followed with:

- If yes [to previous question], are you receiving professional help or treatment for these problems? (yes/no/not applicable)
- If you are currently experiencing psychological problems, is this something you would like professional help or treatment for? (yes/no/not applicable)

These questions were asked at every time point. Binary variables were created with the value ‘1’ if they were receiving treatment or wanted treatment at any time point, and ‘0’ otherwise. Participants who did not answer the question were coded as ‘0’.

History of mental health problems was measured in the early pregnancy questionnaire, which asked whether participants had ever experienced mental health problems. Current physical health problems were also measured at this time point. For both these questions the response options were ‘yes/no/don't know’.

Sociodemographic characteristics were measured by self-report questions based on the England and Scotland Census.

### Procedure

Clinical or research midwives/nurses recruited participants to the MAP cohort in person or remotely when they attended early pregnancy appointments. Women interested in joining the MAP study provided their details and were contacted by the research team, who provided further information, answered questions, obtained written or online informed consent for the cohort study and collected information on whether women consented to be contacted for diagnostic interviews. Women were then sent four self-report questionnaires – three during pregnancy and one postnatally. Before sending questionnaires, checks for serious adverse events were made with NHS sites that participants were attending for their antenatal care. If participants experienced adverse events (e.g. pregnancy loss, stillbirth), the study team checked whether they wanted to continue or withdraw from the study. Questionnaires were completed online or by post, depending on participants preferences. Safeguarding procedures were in place for any participant who scored over the cut-off scores on mental health measures and/or who expressed suicidal intent.

Participants for diagnostic interviews were approached after their questionnaire was returned. Consecutive sampling was used for up to a maximum of 102 women for each time point. Participants who agreed to take part in the diagnostic interview were interviewed within 28 days of returning their questionnaires. Written or recorded and/or verbal informed consent was obtained from all participants. Diagnostic interviews were conducted by psychologists or other clinically qualified members of the research team, who were blind to the results of the questionnaire assessments. Participants were interviewed by telephone and interviews were audio-recorded to check for interrater reliability (96%).

### Analysis

The prevalence of anxiety disorders was examined with descriptive statistics showing the proportion of the sample meeting criteria for diagnosis. Proportions are given for each diagnostic category by time point with respective exact binomial (Clopper–Pearson) confidence intervals. Differences in prevalence across time points were evaluated with logistic regressions, with the dependent variable as ‘diagnosis received’ and the independent variable as ‘time’, which was included as a factor variable. Results are reported as odds ratios with 95% confidence intervals. Degree of comorbidity between anxiety and depression was explored over time points, using logistic regression to assess whether prevalence varied over time for anxiety only, depression only, comorbid anxiety and depression, or no disorder.

The relationship between anxiety disorders and self-reported previous mental health problems, current treatment and desire for treatment were examined with cross-tabulation reporting frequencies and percentages. Analyses were conducted in Stata version 17 for Windows.

## Results

### Prevalence and comorbidity

The mean age of participants was 32.4 years (s.d. 42.4); the majority were married (59.5%) or cohabitating (34.1%), educated to degree level or higher (71.9%) and White British (72.5%). The prevalence of anxiety disorders and comorbid anxiety and depression is shown in Table 1. Over all time points combined, 80 participants met the criteria for an anxiety disorder, giving an overall prevalence of 19.9% (95% CI 16.1–24.1), with the highest prevalence in early pregnancy (25.5%, 95% CI 17.4–35.1) and lowest prevalence in late pregnancy (15.7%, 95% CI 9.2–24.2). For specific disorders, the highest prevalence was found for OCD (8.2%, 95% CI 5.7–11.3), major depressive disorder (6%, 95% CI 3.8–8.7) and GAD (5.7%, 95% CI 3.7–8.4). The lowest prevalence was for post-traumatic stress disorder (2.5%, 95% CI 1.2–4.5) and social anxiety (3.2%, 95% CI 1.7–5.5). Differences in prevalence by time point were only statistically significant for OCD and depression. Participants were significantly less likely to meet criteria for OCD and depression in late pregnancy relative to early pregnancy (OCD: odds ratio 0.26, 95% CI 0.08–0.81, *P* = 0.020; depression: odds ratio 0.17, 95% CI 0.04–0.77, *P* = 0.021). There were no significant differences across time for other diagnostic categories.

**Table 1:** Prevalence of anxiety disorders and comorbid anxiety and depression

	Early pregnancy, *n* = 102% % (95% CI)	Mid-pregnancy, *n* = 99 % (95% CI)	Late pregnancy, *n* = 102 % (95% CI)	Postnatal, *n* = 100 % (95% CI)	Total, *N* = 403 % (95% CI)
Anxiety disorders					
All anxiety disorders	25.5 (17.4–35.1)	19.2 (12.0–28.3)	15.7 (9.2–24.2)	19.0 (11.8–28.1)	19.9 (16.1–24.1)
Generalised anxiety disorder	5.9 (2.2–12.4)	6.1 (2.3–12.7)	4.9 (1.6–11.1)	6.0 (2.2–12.6)	5.7 (3.7–8.4)
Panic disorder	4.9 (1.6–11.1)	4.0 (1.1–10.0)	4.9 (1.6–11.1)	2.0 (0.0–7.0)	4.0 (2.3–6.4)
Agoraphobia	6.9 (2.8–13.6)	2.0 (2.5–7.1)	4.9 (1.6–11.1)	5.0 (1.6–11.3)	4.7 (2.9–7.3)
Specific phobia	4.9 (1.6–11.1)	1.0 (0.0–5.5)	4.9 (1.6–11.1)	3.0 (0.6–8.5)	3.5 (1.9–5.8)
Social anxiety	2.9 (0.6–8.4)	3.0 (0.60–8.60)	3.9 (1.1–9.7)	3.0 (0.6–8.5)	3.2 (1.7–5.5)
Obsessive–compulsive disorder	13.7 (7.7–22.0)	9.1 (4.2–16. 6)	3.9 (1.1–9.7)	6.0 (2.2–12.6)	8.2 (5.7–11.3)
Post-traumatic stress disorder	2.9 (0.6–8.4)	1.01 (0.0–5.5)	5.9 (2.2–12.4)	0 (0–3.6)	2.5 (1.2–4.5)
Major depressive disorder	10.8 (5.5–18.5)	6.1 (2.3–12.7)	2.0 (0.2–6.9)	2.0 (1.6–11.3)	6.0 (3.8–8.7)
Comorbid anxiety and depression					
No diagnosis	72.5 (62.8–80.9)	80.8 (71.7–88.0)	83.3 (74.7–89.9)	80.0 (70.8–87.3)	79.2 (74.8–83.0)
Anxiety only	16.7 (10.0–25.3)	13.1 (7.2–21.4)	14.7 (8.5–23.1)	15.0 (8.6–23.5)	14.9 (11.6–18.7)
Depression only	2.0 (0–6.9)	0.0 (0–3.7)	1.0 (0–5.3)	1.0 (0–5.4)	1.0 (0.2–2.5)
Anxiety and depression	8.8 (4.1–16.1)	6.1 (2.3–12.7)	1.0 (0–5.3)	4.0 (1.1–9.9)	5.0 (3.1–7.6)

Most participants had anxiety disorders only (14.9%, 95% CI 11.6–18.7), 1% had depression only (95% CI 0.2–2.5) and 5% had comorbid anxiety and depression (95% CI 3.1–7.6). Anxiety, depression and comorbidity were highest in early pregnancy, and logistic regression showed the odds of comorbidity were significantly lower in late pregnancy compared with early pregnancy (odds ratio 0.10, 95% CI 0.01–0.82, *P* = 0.032). Differences between time points were not significant for anxiety only or depression only. Lack of significant differences across time may be influenced by low power for analyses across time points.

Table 2 shows the prevalence of anxiety and comorbidity according to whether participants had a history of mental health problems. This shows that the majority of participants with perinatal anxiety or depressive disorders reported a history of mental health problems in the early pregnancy questionnaire. However, it also shows that the majority of participants with a history of mental health problems (65.1%, 95% CI 56.99–72.67%) did not meet the threshold for diagnosis for a perinatal anxiety or depressive disorder.

**Table 2:** Prevalence of current disorders and history of mental health problems

	History of mental health problems, *n* = 152 % (95% CI, *n*)	No history of mental health problems, *n* = 226 % (95% CI, *n*)	Do not know, *n* = 16 % (95% CI, *n*)
No diagnosis	65.1 (56.99–72.67, *n* = 99)	90.7 (86.15–94.16, *n* = 205)	50.0 (24.65–75.35, *n* = 8)
Anxiety only	24.3 (17.75–31.96, *n* = 37)	7.1 (4.10–11.24, *n* = 16)	37.5 (15.20–64.57, *n* = 6)
Depression only	1.97 (0.41–5.66, *n* = 3)	0.44 (0.01–2.44, *n* = 1)	0.0 (*n* = 0)
Anxiety and depression	8.6 (4.63–14.18, *n* = 13)	1.8 (0.48–4.47, *n* = 4)	12.5 (1.55–38.35, *n* = 2)

Overall, 64.6% of participants with a current anxiety and/or depressive disorder had a history of previous mental health problems, 25.6% had no history and 7.3% were not sure. Fewer participants with no history of mental health problems met the threshold for anxiety and/or depressive disorders (9.3%) compared with participants with a history of mental health problems (34.9%). The association between reporting previous mental health problems and meeting the criteria for a diagnosis in the perinatal period was statistically significant (Fisher's exact test *P* < 0.001).

### Treatment for anxiety disorders

The proportion of participants receiving treatment is shown in Table 3. In relation to type of disorder, 15% of those with anxiety disorders, 10% of those with comorbid anxiety and depression and 75% of those with depression were receiving treatment; however, the number of participants with depression only was small (*n* = 4), so this latter finding should be taken with caution.

**Table 3:** Proportion of sample receiving treatment or wanting treatment

	*n*	No diagnosis, % (95% CI, *n*)	Anxiety only, % (95% CI, *n*)	Depression only, % (95% CI, *n*)	Anxiety and depression, % (95% CI, *n*)
Currently receiving professional help or treatment (*n* = 403)					
Yes	24	3.1 (1.51–5.69, *n* = 10)	15.0 (7.10–26.57, *n* = 9)	75.0 (19.41–99.37, *n* = 3)	10.0 (1.23–31.70, *n* = 2)
No	379	96.9 (94.31–98.49, *n* = 309)	85.0 (73.43–92.90, *n* = 51)	25.0 (0.63–80.59, *n* = 1)	90.0 (68.30–98.77, *n* = 18)
Want professional help or treatment (*n* = 403)					
Yes	27	3.1 (1.51–5.69, *n* = 10)	15.0 (7.10–26.57, *n* = 9)	25.0 (0.63–80.59, *n* = 1)	35.0 (15.39–59.22, *n* = 7)
No	376	96.9 (94.31–98.49, *n* = 309)	85.0 (73.43–92.90, *n* = 51)	75.0 (19.41–99.37, *n* = 3)	65.0 (40.78–84.61, *n* = 13)
Whether women who wanted treatment were currently receiving treatment (*n* = 27)					
Receiving treatment	13	60.0 (26.24–87.84, *n* = 6)	55.6 (21.20–86.30, *n* = 5)	100 (2.50–100, *n* = 1)	14.3 (0.36–57.87, *n* = 1)
Not receiving treatment	14	40.0 (12.16–73.76, *n* = 4)	44.4 (13.70–78.80, *n* = 4)	0	85.71 (42.13–99.64, *n* = 6)

A total of 16.7% (*n* = 14) of participants with a diagnosis of anxiety and/or depression were receiving treatment. Interestingly, 3.1% (*n* = 10) of those with no diagnosis were also receiving treatment, which may have been for psychological disorders not included in the diagnostic interviews (e.g. psychosis or neurological disorders).

**Fig. 1:**
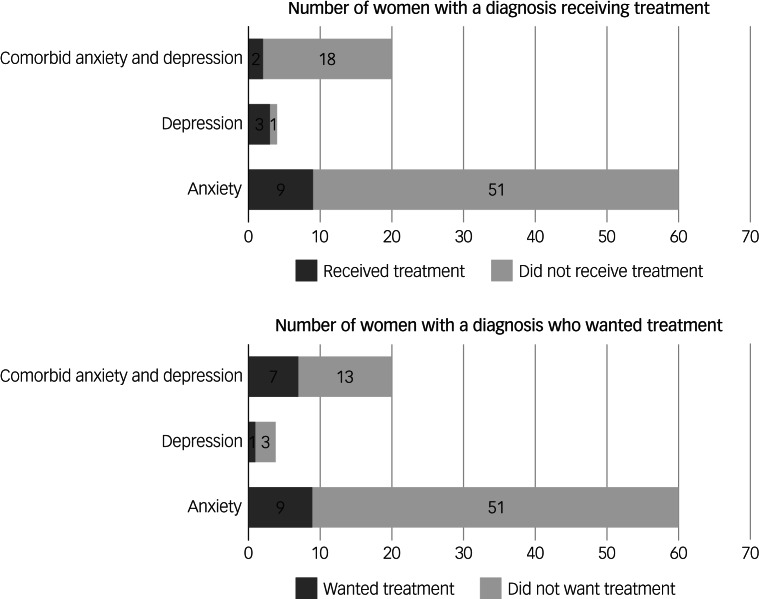
Number of participants with diagnosed anxiety and/or depression who received or wanted treatment (*N* = 84).

A total of 20.2% (*n* = 27) of women with a diagnosis wanted professional help or treatment. This was 15% of those with anxiety disorders and 35% of those with comorbid anxiety and depression. Of those who wanted professional help or treatment, around half (48.1%) were currently receiving treatment, suggesting they wanted more, or different, treatment. Only 14 women who wanted treatment were not receiving treatment (3.7% of the sample), ten of whom had a diagnosis. Figure 1 shows the number of participants with diagnosed anxiety and/or depression who were receiving or wanted treatment.

The majority of women with anxiety or depressive disorders (79.8%) said they did not want treatment. The majority of participants who were not receiving treatment indicated that their psychological issues made things ‘not at all difficult’ or ‘somewhat difficult’ (84.1%), compared with 57.1% of those currently receiving treatment. This difference was statistically significant (Fisher's exact test *P* = 0.03), suggesting that those who perceive their symptoms as having less impact on their day-to-day life are less likely to want or receive treatment.

## Discussion

This study found that one in five women have an anxiety disorder during pregnancy or after birth, and this is highest in early pregnancy, with up to one in four women affected. A key finding is that most women with anxiety disorders did not want professional help or treatment. The reasons for this are unclear. It could be that women felt able to cope with anxiety symptoms by themselves or through other means, such as support from family and peers,^19^ and therefore did not find the symptoms disabling. Alternatively, it could be that the COVID-19 pandemic meant that women were reluctant to have contact with health services or healthcare personnel because of risk of infection. Many other barriers may also have deterred women from wanting professional help or treatment. A review of barriers to accessing perinatal mental health services identified barriers at multiple levels (individual, healthcare professional, interpersonal, organisational, political and societal) across the care pathway, from deciding to consult to receiving care.^20^ Any of these barriers may affect women's desire for treatment and access to care. For example, stigma around perinatal mental health problems and/or lack of confidence in health services may mean that women prefer non-medical support or private routes to treatment.

Thus, there are many reasons why women might not want professional help or treatment. These reasons will depend on the woman, her symptoms and circumstances. It is therefore important not to assume that all women with anxiety want treatment or to automatically refer them to specialist services without exploring whether that is what they want. Clinicians are well-placed to explore this with women, to determine which barriers may need addressing, and whether onward referral is appropriate. Further research is also needed to examine the role of individual and contextual factors in whether women want treatment.

Encouragingly, most of the 20.2% of participants who wanted professional help or treatment were receiving it, with only a very small number of women who wanted treatment not receiving some form of treatment. This suggests that women who want treatment and seek help from health services are able to access treatment.

Although the overall prevalence of anxiety disorders is consistent with previous research,^4^ the prevalence of some disorders was noticeably higher than previously identified. Meta-analyses found that the most frequent anxiety disorders are phobias and GAD,^4^ but in this study, OCD, agoraphobia and panic disorder also had high prevalence rates of between 4 and 8.2%. This higher prevalence of OCD, agoraphobia and panic disorder may be attributable to the study taking place during the COVID-19 pandemic. It is possible, for example, that OCD was elevated because of fear of contagion, or agoraphobia exacerbated by stay-at-home rules (‘lockdown’). It is also possible that the prevalence of OCD was underestimated in previous studies.^3,4^ However, there is substantial heterogeneity in previous research, and the rates found for these disorders are all within the range found previously.^4^

Rates of anxiety reduced through pregnancy, suggesting that initial anxiety may resolve in some women. This and other results from the MAP study suggest that early pregnancy may be the optimal time to screen for perinatal anxiety to identify women with anxiety disorders.^21^ This study also found that two-thirds of women with anxiety disorders had a history of psychological problems, which is consistent with previous research^22^ and clinical recommendations that women's mental health history is asked about during pregnancy.^23^ However, the results also show that the majority of women with a history of mental health problems did not develop an anxiety disorder. Thus, although it is important to ask about mental health history in pregnancy, other factors will also influence whether women with previous mental health problems develop an anxiety disorder.

### Strengths and limitations

This was a population-based cohort study using gold-standard diagnostic interviews at multiple time points, and is the first study to directly examine whether women with anxiety disorders want or receive treatment. However, findings need to be considered in the context of methodological limitations. The number of women who had anxiety disorders in our sample was relatively small, so results should be interpreted cautiously and replicated in larger population-based cohorts. Some anxiety disorders require symptoms to have been experienced for a specified time (e.g. a GAD diagnosis requires 6 months of symptoms), so it is possible that women with new-onset GAD in pregnancy or postpartum were not identified because they did not fulfil this time criterion. Similarly, prevalence rates for depression were low compared with previous research, and the MINI module for post-traumatic stress disorder does not include childbirth-related trauma, so we are unable to draw conclusions about childbirth-related trauma from these findings. Whether participants wanted treatment was asked in the self-report questionnaires, not in the diagnostic interviews, so it is possible that their responses relate to overall mental health, not only the disorder identified in the interview. It is therefore important these areas are examined in future research and methodological limitations addressed.

Finally, the sample was ethnically diverse, but participants had a higher level of education than the general population, and a third reported a history of psychological problems. The sample may therefore not be representative in these respects. As discussed, interviews were conducted during the COVID-19 pandemic, which may have affected prevalence rates for specific disorders such as OCD and agoraphobia, as well as whether participants wanted professional help or treatment.

In conclusion, this study suggests that one in five women have an anxiety disorder during pregnancy or after birth, and this is highest in early pregnancy, with up to one in four women affected at this time. A small proportion of women with anxiety disorders wanted and/or were receiving treatment. Most women did not want professional treatment, and there are many possible reasons for this, including overdiagnosis, individual or contextual factors reducing the desire for treatment, and not wanting contact with health services because of the COVID-19 pandemic. These results highlight the important balance between screening for perinatal mental health problems (because of potential long-term adverse consequences) and not assuming all women want specialist treatment. These findings support the importance of relationship-based care, where individual and contextual barriers/needs can be explored to jointly decide whether a woman would benefit from treatment. A full clinical assessment – during which, women's desire for treatment and treatment preferences can be explored – is important, as well as respecting the preferences of women who do not want treatment. The large majority of women with anxiety disorders had pre-existing mental health problems, which supports clinical recommendations to ask about women's mental health history. Finally, further research is needed to better understand the individual and contextual factors that are important in whether women with anxiety disorders want treatment**.**

## Data Availability

Individual participant-level data are not available, but authors can provide sample-level data and information on request, after publication. The study protocol is available here https://fundingawards.nihr.ac.uk/award/17/105/16.
