# Prevalence and treatment of perinatal anxiety: diagnostic interview study – ERRATUM

**DOI:** 10.1192/bjo.2024.858

**Published:** 2025-01-23

**Authors:** Susan Ayers, Andrea Sinesi, Rose Meades, Helen Cheyne, Margaret Maxwell, Catherine Best, Stacey McNicol, Louise R. Williams, Una Hutton, Grace Howard, Judy Shakespeare, Fiona Alderdice, Julie Jomeen, the MAP Study Team

**Keywords:** Anxiety or fear-related disorders, out-patient treatment, perinatal psychiatry, psychological treatments, observational study, erratum

This article was originally published with the co-author Rose Meades’ name incorrectly given as ‘Rose Meade’. This has now been corrected and this erratum published.

The publisher apologises for the error.
